# Chest Dynamic MRI as Early Biomarker of Respiratory Impairment in Amyotrophic Lateral Sclerosis Patients: A Pilot Study

**DOI:** 10.3390/jcm13113103

**Published:** 2024-05-25

**Authors:** Francesco Barbato, Alessandro Bombaci, Giovanni Colacicco, Giorgia Bruno, Domenico Ippolito, Vincenzo Pota, Salvatore Dongiovanni, Giacomo Sica, Giorgio Bocchini, Tullio Valente, Mariano Scaglione, Pier Paolo Mainenti, Salvatore Guarino

**Affiliations:** 1 Department of Emergency and Urgent Medicine, Stroke Unit, Santa Maria delle Grazie Hospital, 80078 Naples, Italy; francesco.barbato@aslnapoli2nord.it; 2 PhD Program of Neuroscience, Department of Neuroscience “Rita Levi Montalcini”, University of Turin, 10124 Turin, Italy; ale.bombaci@gmail.com; 3 Neurology Unit, IRCSS Policlinico San Donato, 20097 San Donato Milanese, Italy; 4 Department of Neurology, Vita-Salute San Raffaele University, 20132 Milan, Italy; 5 NeuroMuscular Omnicentre (NEMO), Serena Onlus, 20162 Milan, Italy; giovanni.colacicco@centrocliniconemo.it (G.C.); domenico.ippolito@ospedalideicolli.it (D.I.); salvatore.dongiovanni@centrocliniconemo.it (S.D.); 6 Division of Pediatric Neurology, Department of Neurosciences, “Santobono-Pausilipon” Children’s Hospital, 80121 Naples, Italy; giorgiabruno990@gmail.com; 7 Department of Women, Child, General and Specialistic Surgery, University of Campania “Luigi Vanvitelli”, 81100 Caserta, Italy; vincenzo.pota@unicampania.it; 8 Department of Radiology, Monaldi Hospital, Azienda Ospedaliera dei Colli, 80131 Naples, Italy; giorgio.bocchini@ospedalideicolli.it (G.B.); tullio.valente@gmail.com (T.V.); salvatore.guarino@ospedalideicolli.it (S.G.); 9 Department of Medicine, Surgery and Pharmacy, University of Sassari, 07100 Sassari, Italy; mscaglione@uniss.it; 10 Institute of Biostructures and Bioimaging of the National Council of Research (CNR), 80145 Naples, Italy; pierpamainenti@hotmail.com

**Keywords:** motor neuron disease, biomarker, respiratory impairment, non-invasive ventilation, magnetic resonance, diaphragm

## Abstract

**Background:** Amyotrophic lateral sclerosis (ALS) is a neuromuscular progressive disorder characterized by limb and bulbar muscle wasting and weakness. A total of 30% of patients present a bulbar onset, while 70% have a spinal outbreak. Respiratory involvement represents one of the worst prognostic factors, and its early identification is fundamental for the early starting of non-invasive ventilation and for the stratification of patients. Due to the lack of biomarkers of early respiratory impairment, we aimed to evaluate the role of chest dynamic MRI in ALS patients. **Methods:** We enrolled 15 ALS patients and 11 healthy controls. We assessed the revised ALS functional rating scale, spirometry, and chest dynamic MRI. Data were analyzed by using the Mann–Whitney U test and Cox regression analysis. **Results:** We observed a statistically significant difference in both respiratory parameters and pulmonary measurements at MRI between ALS patients and healthy controls. Moreover, we found a close relationship between pulmonary measurements at MRI and respiratory parameters, which was statistically significant after multivariate analysis. A sub-group analysis including ALS patients without respiratory symptoms and with normal spirometry values revealed the superiority of chest dynamic MRI measurements in detecting signs of early respiratory impairment. **Conclusions:** Our data suggest the usefulness of chest dynamic MRI, a fast and economically affordable examination, in the evaluation of early respiratory impairment in ALS patients.

## 1. Introduction

Amyotrophic lateral sclerosis (ALS) is a rare and progressive neurodegenerative disease that recognizes an inherited condition in 10% of cases [1]. The incidence is about 1–3 cases per 100,000 inhabitants per year. Its pathophysiology is unknown [2]. There are different phenotypes, and no effective treatments exist [2]. Diagnosis is purely clinical and neurophysiological [1]. In 20–50% of cases, mild cognitive dysfunction is well documented, and around 5% of patients develop dementia [3]. No effective diagnostic and prognostic biomarkers exist except in particular cases [4]. The average duration of the disease is 2.5 years; in more than half of the cases, death occurs within three years, almost always due to respiratory complications, and less than a quarter of patients survive at least 8 years [5]. It is necessary to coordinate diagnostic and therapeutic interventions in the field of motor, nutritional, and respiratory function to have a positive impact on quality of life and survival. Therefore, the management of this disease requires an integrated multidisciplinary approach that aims to evaluate the timing of invasive and minimally invasive interventions such as the placement of percutaneous endoscopic gastrostomy (PEG), adaptation to non-invasive ventilation (NIV), packaging of a tracheostomy [6]. All these delicate decisions need to be made early with the patient and his family. In this model, the figure of neurologist is decisive in coordination [6].

One of the most adverse prognostic factors in ALS is the presence of respiratory impairment [2]. When respiratory muscles are affected, it leads to restrictive lung disease, primarily caused by the gradual decline in the strength of the diaphragm and intercostal muscles. Initially asymptomatic, this process eventually manifests as respiratory failure symptoms as the damage progresses unavoidably. Since it has been demonstrated that an early start of non-invasive ventilation is fundamental for prolonging survival in ALS patients [7], the exploration of markers indicating pre-clinical respiratory impairment becomes crucial.

Currently, respiratory assessment is based on standard lung function tests, including forced vital capacity (%FVC) in sitting and supine positions, measurement of the volume drops between the standing and supine positions (FVC drop), and estimation of the maximum inspiratory/expiratory pressure (MIP/MEP).

The respiratory muscle involvement begins before spirometry is altered, and seeking a novel earlier biomarker is crucial. Moreover, the above-mentioned tests provide only partial information on the pathophysiological mechanisms of respiratory failure in ALS patients [8]. Therefore, new early biomarkers of respiratory impairment are needed, and while spirometry evaluates overall pulmonary function without distinguishing the specific involvement of different respiratory muscles, chest dynamic magnetic resonance imaging (MRI) can help to specifically study separately the function of the diaphragm, the respiratory muscle most compromised in neuromuscular diseases, and of intercostal muscles [9]. Although it is still difficult to estimate and characterize in depth the function of the diaphragm, the recent advances in MRI technology, such as new and increasingly faster sequences and MRI protocol optimizations, the wide availability of MR tomography, its non-invasiveness and the non-use of ionizing radiation make this tool an excellent opportunity for studying respiratory muscles, especially the diaphragm, and quantifying different aspects of muscle health as already observed by several authors [10,11].

Nevertheless, for some neuromuscular diseases, such as Pompe disease and Duchenne muscular dystrophy (DMD), the impairment of the respiratory muscles has been widely studied with dynamic MRI [9,10,11,12,13,14,15,16,17,18]. To date and to our knowledge, there is only one study, performed by Harlaar et al. [9], reporting a functional evaluation of the diaphragm with dynamic MRI in patients with ALS.

In this study, we aimed to test the usefulness of chest MRI as a biomarker in the identification of early respiratory impairment in ALS patients.

## 2. Materials and Methods

We enrolled 15 patients affected by ALS. These patients received a clinical and neurophysiological diagnosis according to Al Escorial criteria. All patients were assessed using the ALSFRS scale. Other conditions have been excluded through MRI studies of the brain and spine, normal CSF examination, routine biochemical and hematological examinations, and vitamin B12 dosage. Except for one patient, no one reported a history of cigarette smoking; furthermore, no patients were diagnosed with chronic obstructive pulmonary disease (COPD). All MND patients underwent a genetic test for the most common mutations (C9orf72, SOD1, TARDBP, and FUS genes). During the clinical evaluations, it was proposed to perform a chest dynamic MRI. Patients who consented signed the informed consent, which was subsequently archived. As a comparison, 11 healthy non-smoking patients were recruited. The examination of these patients was carried out in the context of a larger radiological protocol to rule out a thymoma.

Forced vital capacity (%FVC) and forced expiratory volume in one second (%FEV1) were measured from flow–volume curves obtained with a spirometer. Non-invasive ventilation has been proposed when PaCO_2_ > 45 mmHg or %FVC < 50% or MIP <60 cmH_2_O, or nocturnal SaO_2_ < 88% for ≥5 consecutive minutes. Peak cough flow (PCF) was measured in unassisted conditions by having the patient cough as hard as possible through a peak flow meter starting from total lung capacity.

All chest MR examinations were performed on a 1.5T MR scanner (Siemens Aera, Erlangen, Germany) using an 18-channel phased array coil. Before the MR examinations, the patients were encouraged to practice maximal inspirations and expirations. Patients were trained to perform the required indications during the radiological examination, as well as what was carried out in respiratory pathophysiology to perform respiratory function tests such as spirometry. The examination was evaluated if suitable by the radiologist. The dynamic sequences performed were part of a routine chest MRI examination. First, a coronal T2 Half Fourier Acquisition Single Shot Turbo spin Echo (HASTE) breath hold scan was obtained. The parameters of the T2 HASTE sequence were an echo time of 95 ms, repetition time of 1400 ms, flip angle of 160°, slice thickness of 7 mm, and acquisition time of 2:00 min. After that, using as reference a T2 HASTE scan including the spine, on both sides, sagittal True Fast Imaging with Steady-state Precession (True FISP) scans passing through the center of the hemidiaphragms were acquired at maximum inspiration and maximum expiration. The parameters of the True FISP sequence were an echo time of 2 ms, repetition time of 383 ms, flip angle of 60°, slice thickness of 5 mm, and acquisition time of 3.9 s.

Using Osirix MD 14.0, the assessment of hemidiaphragm activity was calculated for each lung separately:

- Anterior–posterior lung diameters delta (anteroposterior delta lung on the right [ΔAP_r_] and anteroposterior delta lung on the left [ΔAP_l_]);
- Cranio-caudal lung diameters delta (cranio-caudal delta lung on the right [ΔCC_r_] and cranio-caudal delta lung on the left [ΔCC_l_]).

**Figure 1:**
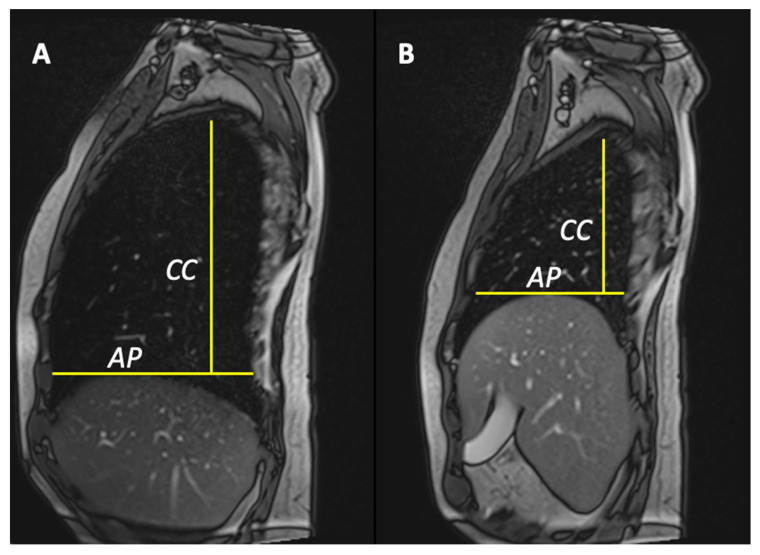
Anterior–posterior lung diameter delta was defined as the differential value between the maximum lung diameters in anteroposterior directions in maximum inspiration (**A**) and maximum expiration (**B**) True FISP scans. Anterior–posterior distance was considered as the distance between anterior and posterior chest wall, tracing a line passing through the top of the hemidiaphragm. Cranio-caudal lung diameters delta was defined as the differential value between the maximum lung diameters in cranio-caudal directions. Cranio-caudal distance was estimated as the distance between the A-P line and the top of the lung.

Anterior–posterior lung diameter delta (ΔAP) was defined as the differential value between the maximum lung diameters in anteroposterior (A-P) directions in inspiration and expiration True FISP scans. A-P distance was considered as the distance between the anterior and posterior chest wall, tracing a line passing through the top of the hemidiaphragm (Figure 1) as previously reported [12]. Cranio-caudal lung diameters delta (ΔCC) was defined as the differential value between the maximum lung diameters in cranio-caudal (C-C) directions in inspiration and expiration True FISP scans. C-C distance was estimated as the distance between the A-P line and the top of the lung (Figure 1). To reduce these four values, in only one, we created two other parameters: the area pulmonary index (PI_area_) and the length pulmonary index (PI_lenght_).

### Statistical Analysis

An exploratory statistical analysis was performed with ANOVA in a way that differentiated both the equality of variances and the distributions of demographic and clinical variables. Continuous variables are presented as averages (SD). Mann–Whitney U test was performed to compare the ALS group with the healthy control group. A linear regression was also performed between each value obtained by MRI (and their recovery indices) and the spirometry values. In addition, a multiple regression analysis was conducted. The significance level was set at 5%. All statistical analyses were performed using SPSS 2022, version 29.0.3.

## 3. Results

In this retrospective study, we evaluated 15 ALS patients and 11 healthy controls.

Of 15 ALS patients, 1 resulted positive for a mutation in the FUS gene. Another patient at subsequent controls was classified as a PLS, in view of Pringle’s criteria [19], considering clinical and neurophysiological data. Therefore, we considered 14 patients, excluding the PLS patients, to make the analysis group homogeneous. In these patients, the mean time to chest MRI from diagnosis was around 19 months, while the median time to tracheostomy was around 7 months. At the time of the examination, no patient was receiving NIV therapy at night, nor had he received a diagnosis of respiratory failure. There were no significant statistical differences between the demographics of the two groups (*p* > 0.05). Comparing both spirometry respiratory parameters (%FVC, %FEV1, and %PCF) and pulmonary measurement at dynamic MRI (ΔAP_r_, ΔAP_l_, ΔCC_r_, and ΔCC_l_) between ALS patients and healthy controls, we observed a statistically significant difference (*p* < 0.001, Table 1).

**Figure 2:**
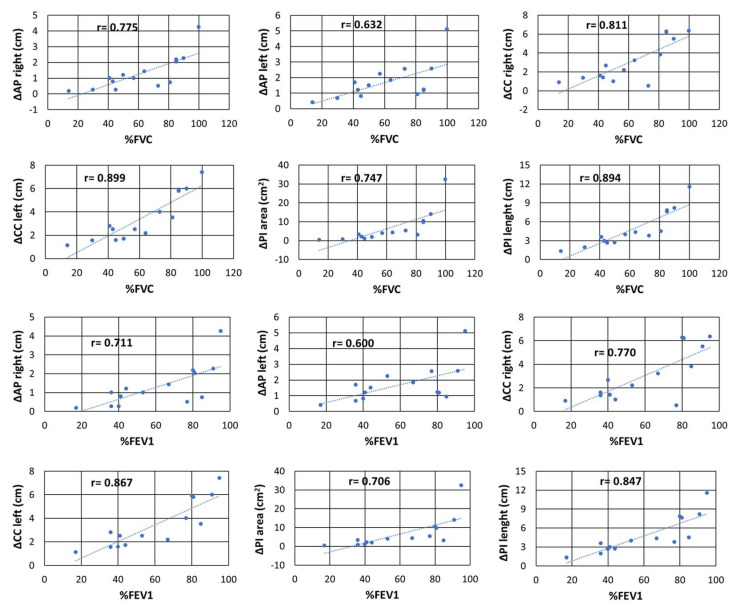
In ALS, we found an important correlation between both %FVC and %FEV1 with all the chest MRI parameters. We observed the highest correlation between %FVC and ΔPI_area_ and ΔPI_length_. Abbreviations: forced vital capacity (FVC); forced expiratory volume in the first second (FEV1); peak flow cough (PFC); anteroposterior delta lung on the right (ΔAP_r_); anteroposterior delta lung on the left (ΔAP_l_); cranio-caudal delta lung on the right (ΔACC_r_); cranio-caudal delta lung on the left (ΔCC_l_); area pulmonary index (ΔPI_area_); length pulmonary index (ΔPI_length_); r = Spearman coefficient.

Moreover, we found a strong correlation between spirometry respiratory parameters and pulmonary measurements at dynamic MRI (especially comparing %FVC with ΔPI_area_ [r = 0.835; *p* < 0.001] and ΔPI_length_ [r = 0.894; *p* < 0.001]); all comparisons are reported in Table 2 and illustrated in Figure 2.

We also led multivariate analysis including sex, age, and Revised Amyotrophic Lateral Sclerosis Functional Rating Scale (ALSFRS-R) at the moment of the measurements, and the strong association between respiratory parameters and dynamic MRI pulmonary measurements was still statistically significant (*p* < 0.001), most of all %FVC with ΔPI_length_ (r = 0.906; *p* = 0.0002). To understand the real potentiality of the dynamic MRI measurements in assessing early respiratory impairments in ALS patients, we selected the sub-group of ALS patients without respiratory symptoms and with normal values of %FVC (greater than 80%), and we compared them with healthy controls. Surprisingly, we observed statistically significant differences in some dynamic MRI measurements (specifically for ΔCC_r_, ΔCC_l_, ΔPI_area_, and ΔPI_length_ [Mann–Whitney, *p* = 0.008], see Supplementary Data), while there were no statistically significant differences in %FVC, %FEV1, and %PCF between ALS patients and healthy controls (*p* > 0.05).

**Figure 3:**
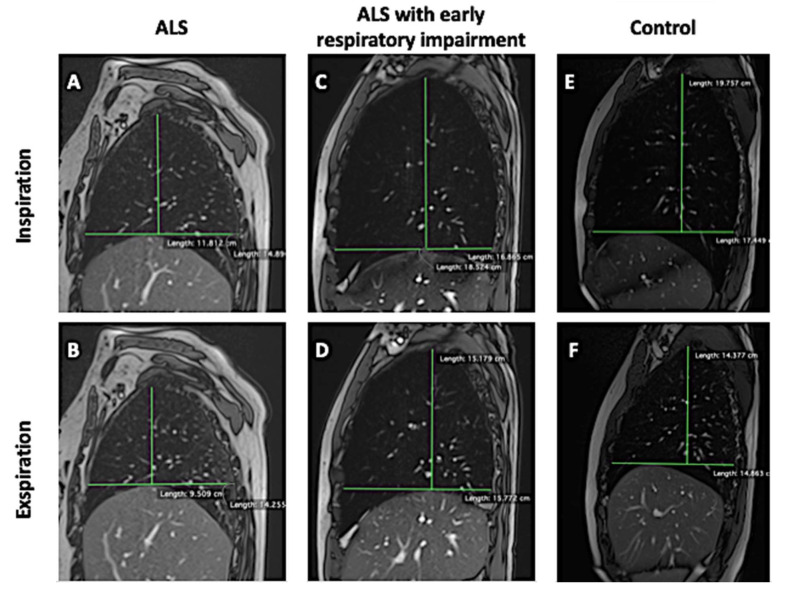
True FISP sagittal scans passing through the center of the right hemidiaphragm acquired at maximum inspiration and maximum expiration showing the differences in AP lung diameters, CC lung diameters, ΔAP_r_, and ΔCC_r_ between an ALS patient (**A**,**B**), an ALS patient with early respiratory impairment (**C**,**D**), and a healthy control (**E**,**F**).

Figure 3 shows in a synoptic and illustrative way differences in AP lung diameters, CC lung diameters, ΔAP_r_, and ΔCC_r_ between an ALS patient (Figure 3A,B), an ALS patient with early respiratory impairment (Figure 3C,D), and a healthy control (Figure 3E,F).

## 4. Discussion

Respiratory muscle function is progressively impaired in neuromuscular disorders affecting respiratory function. Diaphragmatic weakness is the most important component, leading to respiratory failure.

In such disorders, diaphragm ultrasound (US) is a very useful tool because it allows real-time assessment of diaphragmatic contraction, detecting weakness and paralysis, leading to a restrictive respiratory pattern [20,21].

However, there are several limitations to using diaphragm US: US image acquisition and analysis is operator-dependent and requires training [22]; and unsatisfactory visualization of the left hemidiaphragm due to the interposition of gastric air and the limited spleen window [23,24].

An alternative emerging tool for assessing the diaphragmatic function is chest dynamic MRI. In this regard, its potential has already been explored in late-onset Pompe disease and Duchenne muscular dystrophy [DMD].

Several authors found a suitable correlation between chest dynamic MRI data and conventional functional respiratory tests in late-onset Pompe disease. Gaeta et al. showed a strong correlation between pulmonary function tests and diaphragmatic movement area as an expression of diaphragmatic failure [12]. Wens et al. reported that the cranial-caudal movement related to diaphragmatic function was impaired more than the anterior–posterior motions of the anterior chest wall; moreover, they found a suitable correlation between %FVC, %FVC drop, and MRI data, suggesting that both these parameters might be used as an indirect tool for determining diaphragmatic function [13]. Furthermore, Harlaar et al., as well as confirming a correlation between MRI outcomes and pulmonary function tests, demonstrated that dynamic MRI is a sensitive tool for detecting early stages of diaphragmatic weakness in patients with Pompe disease, even when spirometry results are within the normal range [14].

Similar results have been found in studies concerning DMD patients. Mankodi et al. reported that the lung areas at maximal inspiration and expiration were reduced in DMD patients relative to controls, and the change in the lung area between inspiration and expiration correlated with the percent predicted %FVC [15]. Bishop et al. observed that MRI measures of pulmonary function were reduced in DMD and correlated with spirometry data [16]. Pennati et al. reported that structural and functional MRI measurement of diaphragm impairment was highly related to pulmonary function tests, suggesting that MRI could represent a non-invasive tool for the functional and structural assessment of the diaphragm [17]. Barnard et al. found that the sagittal plane lung area was significantly smaller in DMD patients compared to controls at functional residual capacity, tidal inspiration, maximal inspiration, and maximal expiration; moreover, DMD patients also had significantly shorter cranio-caudal thoracic cavity lengths [10].

However, to date and to our knowledge, there is only one study, performed by Harlaar et al. [9], reporting a functional evaluation of the diaphragm with dynamic MRI in patients with ALS. In our study, we aimed to test the usefulness of dynamic MRI as a biomarker in the identification of early respiratory impairment in ALS patients.

The dynamic steady-state free precession MRI sequences are extremely advantageous in ALS patients because they have a short duration and are very fast to acquire.

Our study found a strong correlation between spirometry respiratory parameters (%FVC, %FEV1, and PCF) and chest dynamic MRI measurements (ΔAP_r_, ΔAP_l_, ΔCC_r_, ΔCC_l_, ΔPI_area_, and ΔPI_length_) also in patients with ALS. Moreover, in line with spirometry respiratory parameters, all the chest dynamic MRI measurements were statistically significantly different between ALS patients and healthy controls.

To find a useful index in clinical practice that better resumes the dynamic variations of pulmonary volumes, we created the area pulmonary index (ΔPI_area_) and the length pulmonary index (ΔPI_length_). These two indices best correlate with spirometry values compared to the single-length measurements; furthermore, they can better separate ALS patients from healthy controls.

Another noteworthy datum is the apparent capability of chest dynamic MRI in detecting early respiratory impairment in ALS patients who do not show respiratory symptoms or spirometry alterations. Indeed, within our cohort of ALS patients, we observed that the sub-group comprising individuals without respiratory symptoms and with normal %FVC and %FEV1 values demonstrated a statistically significant reduction in chest MRI parameters. Hence, there is potential for chest MRI to serve as an early biomarker of respiratory impairment. However, further huge, longitudinal, and multicenter studies are required to validate this observation. This is only a preliminary date due to the small number of ALS patients selected in this study. However, this observation is very relevant because the identification of ALS patients with early respiratory impairment is essential for a prompt starting of NIV, which is known to improve survival and quality of life [25].

Moreover, since ALS patients with bulbar impairment present difficulties in performing reliable spirometry because of a lack of perfect adhesion of the lips to the mouthpiece, the use of chest dynamic MRI would be very useful in the evaluation of respiratory muscle impairment in these patients.

Considering the speed and ease of conducting this examination, as well as its cost-effectiveness, we suggest performing it both at diagnosis and throughout the course of the disease. Determining the optimal frequency for repeat examinations during the disease course requires further evaluation.

Limitations of our study are the small number of ALS patients and healthy controls, the cross-sectional design, and the absence of other examinations for the evaluation of respiratory impairment (such as arterial blood gas analysis, the SNIP test, and polysomnography). Therefore, it is currently possible only to hypothesize that this chest dynamic MRI protocol may help detect early respiratory impairment in ALS patients to identify patients at increased risk of developing respiratory failure. If confirmed in greater studies, these data are very important for a better definition of patient prognosis, proper patient stratification that is essential in clinical trials, and the correct selection of patients to undergo an early start of NIV. To confirm our results, a multicenter longitudinal study involving a larger number of ALS patients without respiratory symptoms is mandatory.

## 5. Conclusions

In conclusion, chest dynamic MRI could be a reliable tool for evaluating respiratory impairment in ALS patients. Although data were derived from a small cohort of patients, chest dynamic MRI seems to be useful in evaluating ALS patients without respiratory symptoms or spirometry alterations for the early identification of respiratory muscle impairment. Moreover, in ALS patients with bulbar impairment, this fast and affordable examination seems to be a reliable alternative to spirometry.

## Figures and Tables

**Table 1:** Demographics, respiratory parameters, and chest MRI measurements in ALS patients and healthy controls.

	ALS	HC	*p*_value_
Age	57.8 ± 12.8	56.5 ± 5.7	0.119
Sex (M/F)	5 M; 8 F	5 M; 6 F	0.437 *
Time onset MRI (months ± SD)	25.0 ± 17.6	-	-
Time diagnosis MRI (months ± SD)	19.8 ± 15.5	-	-
FVC (%) ± SD	59 ± 22	97 ± 1.5	8.01 × 10^−7^
FEV1 (%) ± SD	57 ± 22.2	101 ± 4.0	8.01 × 10^−7^
PFC (l/min) ± SD	173 ± 139	459 ± 9.3	1.52 × 10^−5^
ΔAP_r_ (cm) ± SD	1.1 ± 0.7	4.2 ± 0.4	8.01 × 10^−7^
ΔAP_l_ (cm) ± SD	1.4 ± 0.7	4.5 ± 0.9	8.01 × 10^−7^
ΔCC_r_ (cm) ± SD	2.8 ± 2.0	9.0 ± 0.3	8.01 × 10^−7^
ΔCC_l_ (cm) ± SD	3.2 ± 1.7	9.4 ± 0.4	8.01 × 10^−7^
ΔPI_area_ (cm) ± SD	4.6 ± 4.2	40.2 ± 6.4	8.01 × 10^−7^
ΔPI_lenght_ (cm) ± SD	4.3 ± 2.3	13.6 ± 0.8	8.01 × 10^−7^

Abbreviations: forced vital capacity (FVC); forced expiratory volume in the first second (FEV1); peak flow cough (PFC); anteroposterior delta lung on the right (ΔAP_r_); anteroposterior delta lung on the left (ΔAP_l_); cranio-caudal delta lung on the right (ΔCC_r_); cranio-caudal delta lung on the left (ΔCC_l_); area pulmonary index (ΔPI_area_); length pulmonary index (ΔPI_length_); SD (standard deviation). Statistical tests: Mann–Whitney, except for *, where we used χ^2^ test.

**Table 2:** Univariate comparison of respiratory parameters and chest MRI measurements in ALS patients.

	FVC		FEV1		PFC	
	r_s_	*p*_value_	r	*p*_value_	r_s_	*p*_value_
ΔAP_r_	0.775	0	0.71	0	0.61	0.021
ΔAP_l_	0.632	0.02	0.6	0.02	0.45	0.103
ΔCC_r_	0.811	0	0.77	0	0.73	0.003
ΔCC_l_	0.899	0	0.87	0	0.77	0.001
ΔPI_area_	0.747	0	0.71	0.01	0.65	0.013
ΔPI_length_	0.894	0	0.85	0	0.73	0.002
ALSFSRr	0.745	0	0.74	0	0.58	0.032
BMI	−0.087	0.77	-0.1	0.63	0.2	0.4

Abbreviations: body mass index (BMI); forced vital capacity (FVC); forced expiratory volume in the first second (FEV1); peak flow cough (PFC); anteroposterior delta lung on the right (ΔAP_r_); anteroposterior delta lung on the left (ΔAP_l_); cranio-caudal delta lung on the right (ΔCC_r_); cranio-caudal delta lung on the left (ΔCC_l_); area pulmonary index (ΔPI_area_); length pulmonary index (ΔPI_length_); Revised Amyotrophic Lateral Sclerosis Functional Rating Scale (ALSFSRr); Spearman’s correlation coefficient (r_s_).

## Data Availability

Data are contained within the article.

## Supplementary Materials

The following supporting information can be downloaded at: https://www.mdpi.com/article/10.3390/jcm13113103/s1, Table S1: Comparison of chest-mri parameters between healthy controls and ALS patients with normal spirometry examination.
