# Supplementary material for: Chest Dynamic MRI as Early Biomarker of Respiratory Impairment in Amyotrophic Lateral Sclerosis Patients: A Pilot Study
Source: J Clin Med. 2024 May 25;13(11):3103. doi: 10.3390/jcm13113103 (PMC11172785; doi:10.3390/jcm13113103)
Supplement: Supplementary file 1 [file jcm-13-03103-s001.zip › jcm-3003354-supplementary.pdf]

Table S1: Comparison of chest-mri parameters between healthy controls and ALS patients with normal spirometry examination

Phenotype	Age	FVC (%)	ΔAPr (cm)	ΔAPl (cm)	ΔCCr (cm)	ΔCCl (cm)	ΔPI_{area} (cm^2)	ΔPI_{length} (cm)
ALS	56	85	2,2	1,2	6,3	5,9	10,5	7,8
ALS	58	90	2,3	2,6	5,5	6,0	14,0	8,2
ALS	66	80	0,7	0,9	3,8	3,5	3,1	4,5
ALS	58	85	2,0	1,2	6,2	5,8	9,8	7,6
ALS	61	100	4,3	5,1	6,4	7,4	32,4	7,6
HC	51	97	3,7	3,2	8,9	9,1	31,0	12,5
HC	63	95	3,9	4,7	9,2	8,9	38,9	13,4
HC	57	98	3,8	3,1	9,18	9,5	32,2	12,8
HC	48	99	4,1	4,8	8,7	10,2	42,3	13,9
HC	53	98	4,9	5,5	9,5	9,7	50,0	14,8
HC	50	98	3,9	3,4	8,7	8,9	32,1	12,5
HC	59	96	3,8	4,8	9,3	9	39,3	13,5
HC	55	99	4,3	4,7	8,6	9,5	40,8	13,6
HC	66	97	4,7	5,3	9,4	9,8	48,1	14,6
HC	62	97	4,8	4,9	8,7	9,3	43,7	13,9
HC	57	99	4,5	5,1	9,1	9,2	43,9	14,0
**Mann-Whitney Test**	**0.151**	**0,069**	**0,013**	**0,027**	**0,0005**	**0,0005**	**0,003**	**0,0005**

Abbreviations: *Forced Vital Capacity (FVC); Forced Expiratory Volume in one second (FEV1); Peak Flow Cough (PFC); anteroposterior delta lung on the right (*ΔAP_r*); anteroposterior delta lung on the left (*ΔAP_l*); cranio-caudal delta lung on the right (*ΔCC_r*); cranio-caudal delta lung on the left (*ΔCC_l*); area pulmonary index (*ΔPI_{area}*); length pulmonary index (*ΔPI_{length}*).*
